# Supplementary material for: Strains on the human femur after revision total knee arthroplasty: An in vitro study using digital image correlation
Source: PLoS One. 2024 Jun 13;19(6):e0305387. doi: 10.1371/journal.pone.0305387 (PMC11175519; doi:10.1371/journal.pone.0305387)
Supplement: Attachment — Submitted filename: Response_to_Reviewers.docx [file pone.0305387.s003.docx]

Pawel Klosowski, D.Sc.
Academic Editor
PLOS ONE

Aesculap AG
Research & Development

Postfach 40
78501 Tuttlingen
Germany

e-mail: thomas.grupp@aesculap.de
http://www.bbraun.com

Your reference	Our reference	Tel. +49/74 61/95-	Fax +49/74 61/95-	Date

PEE-TGR	2667	382667	February 10, 2024

**Revised Manuscript for publication as Research Article** **in Public Library of Science**

Dear Dr. Klosowski,

## Thank you for giving us the opportunity to submit a revised draft of the manuscript “Strains on the human femur after revision total knee arthroplasty: An in vitro study using digital image correlation” to PLOS ONE.

## We appreciate the time and effort that you and the reviewers have dedicated to providing your valuable feedback on our manuscript. We appreciate the reviewers´ insightful comments and are confident that they contributed to increasing the quality of our manuscript.

## We are pleased that we were able to address all suggested changes. We have highlighted the changes within the manuscript.

## In the following, you can find the reviewers´ comments with a point-by-point response in italics as well as the revised conflict of interest statement.

Thank you for your consideration.

Yours sincerely,

Elisabeth Sporer

Revised Conflict of Interest Statement

Three of the authors (EMS, CS, TMG) are employees of Aesculap AG, a manufacturer of orthopedic implants.

RT and AG are paid consultants for Aesculap AG. AG is receiving royalties from

Aesculap AG and is an unpaid consultant for DePuy Synthes. He is member of the Austrian Orthopaedic Society and of ”AE – Arbeitsgemeinschaft Endoprothetik”. RT is receiving royalties from Conformis and is a paid consultant for this company. RT has stock or stock options in OnPoint Surgical and receives support from Conformis and ZimmerBiomet as Principal Investigator. TMG is scientific member of the working group „Evaluations & Studies“ of the German National Joint Registry „Endoprothesenregister Deutschland“ (EPRD), Advisory Board Member of the EU Consortium SPINNER “Next generation of repair materials & techniques for spine surgery” and Chair of working group I “Introduction of Innovations” of the ”European Federation of National Associations of Orthopaedics and Traumatology” (EFORT) “Implant & Patient Safety Initiative”. This does not alter our adherence to PLOS ONE policies on sharing data and materials.

Comments

Journal requirements:

1. When submitting your revision, we need you to address these additional requirements.

Please ensure that your manuscript meets PLOS ONE's style requirements, including those for file naming. The PLOS ONE style templates can be found at 
https://journals.plos.org/plosone/s/file?id=wjVg/PLOSOne_formatting_sample_main_body.pdf and 
<https://journals.plos.org/plosone/s/file?id=ba62/PLOSOne_formatting_sample_title_authors_affiliations.pdf>.

*Response: We have updated the style of the manuscript and adapted the figure file naming and the style of the headings to PLOS ONE requirements.*

2. Please provide additional details regarding participant consent. In the ethics statement in the Methods and online submission information, please ensure that you have specified (1) whether consent was informed and (2) what type you obtained (for instance, written or verbal, and if verbal, how it was documented and witnessed). If your study included minors, state whether you obtained consent from parents or guardians. If the need for consent was waived by the ethics committee, please include this information.

If you are reporting a retrospective study of medical records or archived samples, please ensure that you have discussed whether all data were fully anonymized before you accessed them and/or whether the IRB or ethics committee waived the requirement for informed consent. If patients provided informed written consent to have data from their medical records used in research, please include this information.

*Response: We agree that ensuring adequate informed consent of the participants is important. In the ethical vote for this study this point is clearly addressed: Only specimens from so-called cadaver donors are used for this study. These individuals have kindly and voluntarily donated their bodies (to the Centre for Anatomy and Cell Biology - Medical University of Vienna) during their lifetime per written consent for the purposes of pre- and postgraduate teaching and scientific research. The anonymity of the study participants is strictly maintained and only age and gender are recorded. These two pseudonymised data are passed on to the project partner (B.Braun-Aesculap).*

3. Thank you for stating the following in the Competing Interests section: 
[I have read the journal's policy and the authors of this manuscript have the following competing interests:
Three of the authors (EMS, CS, TMG) are employees of Aesculap AG, a manufacturer of orthopedic implants.
RT and AG are paid consultants for Aesculap AG. AG is receiving royalties from 
Aesculap AG and is an unpaid consultant for DePuy Synthes. He is member of the Austrian Orthopaedic Society and of ”AE – Arbeitsgemeinschaft Endoprothetik”. RT is receiving royalties from Conformis and is a paid consultant for this company. RT has stock or stock options in OnPoint Surgical and receives support from Conformis and ZimmerBiomet as Principal Investigator. TMG is scientific member of the working group „Evaluations & Studies“ of the German National Joint Registry „Endoprothesenregister Deutschland“ (EPRD), Advisory Board Member of the EU Consortium SPINNER “Next generation of repair materials & techniques for spine surgery” and Chair of working group I “Introduction of Innovations” of the ”European Federation of National Associations of Orthopaedics and Traumatology” (EFORT) “Implant & Patient Safety Initiative”.].

Please confirm that this does not alter your adherence to all PLOS ONE policies on sharing data and materials, by including the following statement: ""This does not alter our adherence to  PLOS ONE policies on sharing data and materials.” (as detailed online in our guide for authors http://journals.plos.org/plosone/s/competing-interests).  If there are restrictions on sharing of data and/or materials, please state these. Please note that we cannot proceed with consideration of your article until this information has been declared.

Please include your updated Competing Interests statement in your cover letter; we will change the online submission form on your behalf.

*Response: You can find the revised conflict of interest statement including the confirmation of our adherece to all PLOS ONE data sharing policies on the previous page.*

4. When completing the data availability statement of the submission form, you indicated that you will make your data available on acceptance. We strongly recommend all authors decide on a data sharing plan before acceptance, as the process can be lengthy and hold up publication timelines. Please note that, though access restrictions are acceptable now, your entire data will need to be made freely accessible if your manuscript is accepted for publication. This policy applies to all data except where public deposition would breach compliance with the protocol approved by your research ethics board. If you are unable to adhere to our open data policy, please kindly revise your statement to explain your reasoning and we will seek the editor's input on an exemption. Please be assured that, once you have provided your new statement, the assessment of your exemption will not hold up the peer review process.

*Response: Yes - all data are fully available without restriction.*

*All authors of this study agree to this.* *All relevant data are within the manuscript and its Supporting Information files.*

5. Please include the reference section of your manuscript.

*Response: The reference section is included in the manuscript.*

6. We note that Figure 1, 2, 6, 7, and 8 in your submission contain copyrighted images. All PLOS content is published under the Creative Commons Attribution License (CC BY 4.0), which means that the manuscript, images, and Supporting Information files will be freely available online, and any third party is permitted to access, download, copy, distribute, and use these materials in any way, even commercially, with proper attribution. For more information, see our copyright guidelines: http://journals.plos.org/plosone/s/licenses-and-copyright.
 
We require you to either (1) present written permission from the copyright holder to publish these figures specifically under the CC BY 4.0 license, or (2) remove the figures from your submission:
 a. You may seek permission from the original copyright holder of Figure 1, 2, 6, 7, and 8 to publish the content specifically under the CC BY 4.0 license. 
 
We recommend that you contact the original copyright holder with the Content Permission Form (http://journals.plos.org/plosone/s/file?id=7c09/content-permission-form.pdf) and the following text:
“I request permission for the open-access journal PLOS ONE to publish XXX under the Creative Commons Attribution License (CCAL) CC BY 4.0 (http://creativecommons.org/licenses/by/4.0/). Please be aware that this license allows unrestricted use and distribution, even commercially, by third parties. Please reply and provide explicit written permission to publish XXX under a CC BY license and complete the attached form.”
 Please upload the completed Content Permission Form or other proof of granted permissions as an ""Other"" file with your submission. 
 In the figure caption of the copyrighted figure, please include the following text: “Reprinted from [ref] under a CC BY license, with permission from [name of publisher], original copyright [original copyright year].”
 
b. If you are unable to obtain permission from the original copyright holder to publish these figures under the CC BY 4.0 license or if the copyright holder’s requirements are incompatible with the CC BY 4.0 license, please either i) remove the figure or ii) supply a replacement figure that complies with the CC BY 4.0 license. Please check copyright information on all replacement figures and update the figure caption with source information. If applicable, please specify in the figure caption text when a figure is similar but not identical to the original image and is therefore for illustrative purposes only.

*Response: Thank you for this input. All pictures included in this study were taken by the author, Dr. med. Elisabeth Sporer, and have not been published before. There are no third party figures. All authors agree with publication of the figures.*

Additional Editor Comments:

According to the Reviewers opinion it is necessary to emphasise the novelty of the research. The results presented in the paper should be compared with the results of other investigators. Additionally, other minor remarks are indicated in the reports should be considered.

*Response: Thank you for your valuable input. We are happy that we were able to adress your recommendations and further improve the manuscript. We were able to compare our results to two previous studies (Kim et al. 2008, Completo et al 2012). The main novelty of our study is the experimental in-vitro design and the focus on the human femur. Completo et al. also used an in-vitro study design but analyzed tibiae. Our study is the first one showing strain concentrations at the tip of the femoral stem in revision total knee arthroplasty. We have tried to improve the manuscript by emphasizing the novely of the research.*

Reviewers' comments:

**Comments to the Author**

Reviewer #1: Dear Authors,

Thank you for your contribution to the field with this insightful study. However, I would like to request more information regarding the choice of the two specific loading scenarios - stair climbing and rising from a chair - employed in your experiments.

While these scenarios are undoubtedly relevant to the daily activities of patients undergoing revision total knee arthroplasty (TKA), the rationale behind selecting specifically these two scenarios over others is not clearly articulated in the manuscript. Understanding the reasoning for this selection is crucial, as it provides insight into the applicability and relevance of your findings to the broader context of post-operative patient activities.

Could you please elaborate on the following points:

The criteria or considerations that led to the selection of these particular scenarios.
Whether these scenarios are representative of the most common or most challenging activities encountered by patients post-revision TKA.
If there were any other scenarios considered during the planning phase of your study, and if so, why they were ultimately not included.

*Response: Thank you for this insightful comment! We totally agree that the selection of adequate loading scenarios is crucial.* *Though the pain is described as activity related, there is no information in the literature about specific activities that provocate End-of-Stem Pain (Barrack et al., 1999, Barrack et al., 2004). Therefore, we have decided for two scenarios that are part of the daily activities of patients. We investigated one scenario with high bending moments at the tip of the stem (ChairRise90°) and one scenario with predominantly axial forces (Stairs20°). By choosing these loading scenarios we were able to analyze both biomechanical situations. We have added this information to the manuscript.*

The selection criteria for the femurs used in your experiments, including any specific characteristics or parameters that were considered.

*Response: There were no specific inclusion criterias for the used donor bones except for no former surgery of the bone, no obviouse bone disease and no preexisting knee implantat.*

Clarifying these aspects would greatly enhance the understanding of the context and significance of your findings, thereby contributing to a more comprehensive interpretation of your research.

Reviewer #2: The presented problem is interesting, the experimental research was performed rigorously and described well.

However, the results regarding strains concentration and its character are rather obvious, and also expected by the Authors. Therefore, it is difficult to find novelty in the presented research.

*Response: We agree that we expected to find strain concentrations at the tip of the stem. Nevertheless, there are no previous studies that were able to measure femoral strain concentrations. The finding has important implications for the future. It will allow for in-vitro development of new stem designs with a better biomechanical performance by using different materials or stem designs such as fluted stems, for example.*

Furthermore, the final conclusion that DIC method is “valuable method for future tests of biomechanical characteristics in different applications” is rather a general statement, as a lot of up-to-date biomechanical studies uses the DIC measurements already. Thus, application of the DIC measurements cannot be taken as the main novelty of the paper.

*Response: We totally agree with the reviewer that DIC method has become an established method for strain measurement. Therefore, we have excluded the statement cited above from the manuscript.*

Finally, in my opinion, the authors did not find satisfactory biomechanical correlation between the end-of-stem pain and the obtained results. The presented research seems to be rather a pilot study and more numerical and clinical analysis should be added to make it comprehensive.

*Response: Thank you for your feedback. We agree that there is more research necessary and that the correlation between End-of-Stem Pain and strain concentrations at the tip of the stem cannot be shown in our study due to the preclinical study design. Nevertheless, our study provides an important first step for the understanding of End-of-Stem Pain and builds the necessary basis for further research. Now that strain concentrations at the tip of the stem have been shown, future studies can focus on different materials and stem designs and their effect on surface strains.*

Additional remarks:
Fig. 4 is of poor quality, thus making the analysis of the results difficult.

*Response: We have improved the resolution of Fig. 4.*

How was the surface of the bone prepared before applying aerosol spray and the DIC measurements?

*Response: We approve of the reviewer´s suggestion of adding more information about bone preparation. Soft tissue was removed carefully using a bone curette, without damaging the surface of the bone. The bone was then cleaned using isopropanol before application of aerosol spray. We have added this information to the manuscript.*


Reconsider, if the specimens with preexisting fracture should be included in the result section, as this fracture suggests that the stem was implanted improperly and therefore can affect the results.

*Response: We completely share the reviewer´s opinion that the preexisting fractures are important to keep in mind! We have discussed the reviewer´s comment intensely. On the one hand, preexisting fractures might influence the results. On the other hand, the implantation was performed following the recommended surgical technique and there was no reasonable suspicion for improper implantation. The small longitudinal fractures might also occure in some patients and may not be obvious on standard radiological imaging. We have therefore decided to include these specimens in the results section.*
