# Supplementary material for: Strains on the human femur after revision total knee arthroplasty: An in vitro study using digital image correlation
Source: PLoS One. 2024 Jun 13;19(6):e0305387. doi: 10.1371/journal.pone.0305387 (PMC11175519; doi:10.1371/journal.pone.0305387)
Supplement: Attachment — Submitted filename: Response_to_Reviewers.docx [file pone.0305387.s004.docx]

Your reference	Our reference	Tel. +49/74 61/95-	Fax +49/74 61/95-	Date

PEE-TGR	2667	382667	May 08, 2024

**Revised Manuscript for publication as Research Article** **in Public Library of Science**

Dear Editor, Dear Reviewers,

Thank you for your valuable feedback on our manuscript.

We have addressed all suggested changes.

In the following, you can find the reviewers´ comments with a point-by-point response in italics.

Thank you for your consideration.

Yours sincerely,

Dr. med. Elisabeth Sporer

Reviewers´ commernts:

Data Analysis Methodology: Regarding the method of measuring surface deformation using Digital Image Correlation (DIC), I would appreciate a more detailed technical explanation and a thorough discussion of its efficacy. Specifically, we need more information on the accuracy and reproducibility of measurements achieved through DIC, as well as the advantages of this method compared to existing techniques.

*Strain gauges have been considered the gold standard for strain measurement for a long time, but they only offer strain measurement at discrete points. DIC is a non-contact method to measure strains and it allows for a full-field strain analysis. In a study by Correa et al. (Correa et al. 2018), correlation between strain measurement using strain gauges and DIC has been shown.* *With the system used in our study an approximated accuracy for strain of 0.1% was achieved for both in-plane and out-of-plane measurement. The reproducibility is approximately +/- 0.2%. We have added this information to the manuscript.*

Analysis of Fracture Patterns: While your manuscript presents results on fracture patterns post-excessive loading, it would greatly benefit from a more explicit explanation of how these patterns contribute to the understanding of the biomechanical mechanisms of End-of-Stem Pain. Additionally, further information is needed on quantitative approaches to fracture pattern analysis and how these results should be interpreted clinically.

*Until now, the amount of biomechanical influence of the stem in knee arthroplasty has still been unknown. The specific fracture pattern found in this study suggests that there is a high biomechanical impact of the stem. It is also still unclear why only certain patients suffer from End-of-Stem Pain whereas others do not experience End-of-Stem Pain. In our study, only in some specimens overcritical load led to the stem pattern fracture. This may suggest that the biomechanical influence of the stem is higher in some patients than in others. This may explain why only some patients suffer from End-of-Stem Pain and underlines the importance of individualized selection of the right implant. We have added this to the discussion of our manuscript.*

I am still interested if there are any other studies regarding strains on the bone and their correlation with any pain. Do you know what level of strain results in pain? Was it any time before tested or checked? Maybe on the other bones or tissues? In my opinion, a discussion on this point is worth to be included in the paper, which could make your research more comprehensive. If such details are not available, they should be strongly highlighted or explained in the text.

*Unfortunately**, to our knowledge there are no studies that report which level of strain results in pain, neither at the human femur nor at other bones*. *We strongly agree that further studies are required in this field.*

In the discussion section, you mentioned about similarities between your results of the obtained strain levels with the outcomes of other researchers. It would enhance the discussion if you put more detailed information about the comparison of these results, e.g. - what was the highest strain level in your research versus the highest one obtained by others? what was the range of strains obtained by you and the others? does this range change due to the type of bone analyzed or the method of measurement applied? More numbers will make the discussion more clear and informative. In my opinion, statements, that “the results are similar” is too general.

*Thank you for this feedback, we agree that discrete numbers help to interpret the results. We have added a paragraph precisely comparing the results of both studies to our manuscript.*
